# B.R.E.A.S.T. Study protocol: Benefits of R-TEP EMDR protocol in Addressing diStress and Trauma in breast cancer patients

**DOI:** 10.3389/fpsyg.2025.1759849

**Published:** 2026-01-20

**Authors:** Livia Emma Ligorio, Alessandro Alberto Rossi, Rebecca Sala, Francesca Pesavento, Flavia Musco, Stefania Mannarini, Roberto Bollina

**Affiliations:** 1 Clinical Psychology Unit, ASST Rhodense, Garbagnate Hospital, Garbagnate, Italy; 2 Department of Philosophy, Sociology, Education, and Applied Psychology, Section of Applied Psychology, University of Padova, Padova, Italy; 3 Center for intervention and Research on Family Studies – CIRF – Department of Philosophy, Sociology, Education, and Applied Psychology, Section of Applied Psychology, University of Padova, Padova, Italy; 4 Breast Surgery Unit, ASST Rhodense, Garbagnate Hospital, Garbagnate, Italy; 5 Medical Oncology Unit, ASST Rhodense, Garbagnate Hospital, Garbagnate, Italy

**Keywords:** breast cancer, EMDR R-TEP, eye movement desensitization and reprocessing, post-traumatic stress disorder (PSTD), psychological distress, psycho-oncology, randomized controlled trial

## Abstract

**Background:**

Breast cancer diagnosis and surgical treatment can be experienced as traumatic events, triggering psychological distress including anxiety, depression, and post-traumatic symptoms. The Recent Traumatic Episode Protocol (R-TEP), an early EMDR intervention, has shown efficacy in reducing psychological symptomatology following recent traumatic events.

**Objectives:**

This protocol describes a randomized controlled trial designed to assess the efficacy of the EMDR R-TEP protocol in primarily reducing psychological distress and post-traumatic symptoms in breast cancer patients undergoing surgery, with secondary focus on emotion regulation, hopelessness, body image and global psychological symptomatology.

**Methods:**

A prospective, longitudinal, single-blind randomized controlled trial will be conducted at the Breast Unit of ASST Rhodense (Milan, Italy). A minimum of 122 female patients, accounting for 20% attrition, with first breast cancer diagnosis scheduled for surgery will be randomized into three groups: treatment as usual (TAU), TAU plus psychoeducational intervention, and TAU plus EMDR R-TEP. Assessments will be conducted at baseline (pre-surgery), post-surgery/pre-psychological intervention, post-psychological intervention, and at 3-month follow-up. Primary outcomes include the Psychological Distress Inventory-Revised and Post-Traumatic Symptom Questionnaire.

**Discussion:**

This protocol provides the methodological framework for generating evidence on EMDR R-TEP as an early psychological intervention for breast cancer patients.

## 1 Introduction

Breast cancer is one of the most common cancers affecting women worldwide, with significant physical and psychological implications for patients (AIRTUM Working Group, 2024). The psychological condition of oncological patients has been shown to significantly influence the course of medical treatments and adherence to therapeutic protocols (Theofilou and Panagiotaki, 2012; Faretta and Borsato, 2016; Dinapoli et al., 2019). Psychological symptomatology and psychological distress appears to have a substantial negative impact on patients’ overall well-being, deteriorating their quality of life considerably. In particular, breast cancer presents unique psychological challenges related to femininity, body image, and fear of recurrence, making psychological support essential (Koçan and Gürsoy, 2016; Pierrisnard et al., 2018). Furthermore, beyond these challenges, cancer diagnosis and treatment may also constitute a traumatic experience, particularly when perceived as life-threatening (Leano et al., 2019; Portigliatti Pomeri et al., 2021).

### 1.1 Psychological trauma and oncological illness

Literature suggests that cancer diagnosis and surgical treatment can trigger post-traumatic symptoms (Smith et al., 1999; Cordova et al., 2017; Banik et al., 2019; Panzeri et al., 2023) such as intrusion, avoidance, and hyperarousal (Bates et al., 2023; Rossi et al., 2024a), as well as a broader pattern of psychological distress that includes anxiety, depression (Fischer and Wedel, 2012; Lo-Fo-Wong et al., 2016), and adjustment disorders. Furthermore, surgical interventions such as mastectomy or quadrantectomy can carry additional traumatic implications, affecting body image and the related psychological sphere (Maguire, 1985; Rossi et al., 2022, 2024a).

These traumatic responses can be attributed to multiple factors. Indeed, patients face a direct confrontation with mortality, experiencing fears regarding treatment outcomes and survival that exacerbate emotional distress (Abbey et al., 2015; Swartzman et al., 2017) – often intensified by fear of recurrence contributing to chronic anxiety and PTSD-related symptoms (Mehnert et al., 2009). Cancer also entails a profound loss of control over one’s body and fate, particularly in breast cancer where mastectomy physically alters the body and instills feelings of vulnerability and helplessness (Mehnert et al., 2009; Danhauer et al., 2013). Furthermore, uncertainty surrounding prognosis and treatment efficacy creates persistent trauma-related distress, with patients reporting intrusive thoughts and hyperarousal linked to the unpredictability of their condition (Andrykowski and Cordova, 1998; Mehnert and Koch, 2007). Finally, the invasiveness of medical procedures represents a continuous source of traumatic exposure throughout the treatment trajectory (Cordova et al., 2017).

Additionally, several psychological factors have been associated with post-traumatic symptoms – potentially exacerbating and reinforcing the traumatic response. Emotion dysregulation has been linked to maladaptive coping strategies such as avoidance and denial, which may exacerbate PTSD symptoms (Oniszczenko and Laskowska, 2014; Davis et al., 2023; Ouhmad et al., 2023). Similarly, hopelessness stemming from perceived lack of control over health outcomes (Gidron et al., 2001; Pérez et al., 2014), low self-esteem (Schwabish, 2011), and lack of social support (Andrykowski and Cordova, 1998; Zhang et al., 2025) have all been associated with higher post-traumatic symptomatology. These psychological consequences extend beyond patients, affecting also caregivers (Benedict et al., 2025). Furthermore, PTSD symptoms may emerge not only immediately following diagnosis but also as delayed-onset presentations (Elklit and Blum, 2011).

### 1.2 Psychological distress and quality of life

In oncological settings, psychological distress (i.e., distress) has been conceptualized as the full amount of cancer-related emotions and feelings experienced by patients that may affect their ability to cope with cancer itself (Holland and Bultz, 2007; Compen et al., 2018). Moreover, given its pervasive impact on both psychological and physical health, distress could be considered as the “sixth vital sign” in oncology care (Bultz and Carlson, 2006; Grassi and Riba, 2012; Riba et al., 2019).

Distress has been consistently associated with significant impairments across multiple domains of functioning, affecting physical, emotional, and social well-being, and overall quality of life (Derogatis et al., 1983; Maguire, 1985; Fallowfield et al., 2001; Riba et al., 2019; Muzzatti et al., 2020), reduced adherence to treatment and self-management (Newell et al., 1998; Partridge et al., 2002; Spiegel and Giese-Davis, 2003; Granek et al., 2019), and poorer survival outcomes (Trask et al., 2002; Kwak et al., 2013).

The prevalence of psychological distress varies considerably across cancer types, with breast cancer patients reporting among the highest levels (Kaasa et al., 1993; Portenoy et al., 1994; Vodermaier et al., 2009; Holland and Alici, 2010; Mehnert et al., 2018) — nearly 41% experience significant distress, with anxiety, depression, and hopelessness being the most common presentations (Zabora et al., 2001; Herschbach et al., 2004; Tsaras et al., 2018). In breast cancer, quality of life impairments have been documented across physical, emotional, social, and sexual functioning domains (Pierrisnard et al., 2018; Muzzatti et al., 2020; Pezzolato et al., 2023). Furthermore, treatments such as chemotherapy and surgery may significantly affect quality of life — particularly fatigue, body image, sexual functioning, and cognitive functioning — though partial recovery is often observed within the first year post-treatment (Ganz et al., 1996; Carreira et al., 2021; Biparva et al., 2023; Heidary et al., 2023).

Moreover, longitudinal studies indicate that at least one-third of patients report persistently elevated distress levels, which may remain stable or increase as the disease progresses (Carlson and Bultz, 2003; Sellick and Edwardson, 2007), with a substantial proportion continuing to experience clinically significant anxiety, depression, and cancer-related distress months to years following diagnosis (Lo-Fo-Wong et al., 2016; Mehnert et al., 2018; Rossi et al., 2022).

### 1.3 Body image and femininity in breast cancer

The impact of mastectomy on body image represents a critical dimension of the psychological burden experienced by breast cancer patients (Türk et al., 2018). For many women, breasts symbolize femininity, sexuality, beauty, and motherhood, and their removal through mastectomy can result in profound disturbances to body image and self-concept (Fang et al., 2013; Koçan and Gürsoy, 2016). Research has documented that negative body image following mastectomy includes dissatisfaction with appearance, perceived loss of femininity and body integrity, reluctance to look at one’s body, feeling less sexually attractive, self-consciousness about appearance, and dissatisfaction with surgical scars (Koçan and Gürsoy, 2016; Latifee et al., 2025).

Studies have shown that mastectomy can be experienced as a sense of mutilation and diminished self-worth, threatening perceptions of femininity and potentially causing more psychological trauma than the cancer diagnosis itself (Fang et al., 2013; Türk et al., 2018). Body image distress is associated with additional complications including reduced quality of life, depression and anxiety symptoms, social isolation, and impairments in sexual functioning (Weingarden et al., 2022; Latifee et al., 2025). These psychological impacts may persist long after treatment completion, affecting social reintegration and intimate relationships, with some studies suggesting that adaptation may take up to 2 years following surgery (Izydorczyk et al., 2018).

### 1.4 Psychological support needs

Moreover, thanks to advances in treatment, many breast cancer patients survive longer, creating an increasing need for interventions aimed at improving psychological condition and emotional wellbeing (Andersen et al., 2008; Mannarini et al., 2018; Panzeri et al., 2024). Research has shown that intrusive thoughts related to breast cancer are associated with enduring elevations in behavioral symptoms and impaired quality of life in the year following treatment, potentially serving as a risk factor for poor long-term outcomes (Dupont et al., 2014). This underscores the importance of early psychological intervention to prevent the development of chronic distress and facilitate positive adjustment.

In recent years, the scientific community has increasingly emphasized the importance of providing adequate psychological support to oncological patients to address and appropriately manage this clinical picture (AIRTUM Working Group, 2024) – both related to cancer diagnosis and the subsequent surgical intervention for its removal.

### 1.5 Psychoeducational interventions in breast cancer

In addition to psychological support interventions, numerous studies have highlighted the role of psychoeducational interventions in reducing anxiety and depressive symptoms associated with cancer (Al-Alawi et al., 2022). Psychoeducational interventions have emerged as a promising approach for supporting women with breast cancer, providing patients with information, emotional expression opportunities, coping skills, and support (Al-Alawi et al., 2022; Mustikaningsih et al., 2024). Such interventions are configured as fundamental elements to prepare patients for symptom management, post-operative recovery, promote self-care practices, increase perception of control, and manage emotions (Al-Alawi et al., 2022). Furthermore, the reflective components of psychoeducation may promote self-awareness, emotion regulation, and self-esteem (Mannarini, 2009, 2010; Charalampopoulou et al., 2020; Ghanbari et al., 2021; Sebri et al., 2021; Wojtyna et al., 2023), which are particularly relevant for women facing challenges following breast cancer diagnosis and surgery (Morales-Sánchez et al., 2021; Setyowibowo et al., 2022).

Psychoeducational interventions have demonstrated efficacy in reducing psychological distress, anxiety, and depressive symptomatology among breast cancer patients. A comprehensive meta-analysis found that psychoeducation significantly reduced anxiety levels and improved quality of life in breast cancer patients (Setyowibowo et al., 2022). Another systematic review and meta-analysis found that psychoeducational interventions significantly decreased psychological distress levels in women with breast cancer diagnosis compared to control groups (Al-Alawi et al., 2022). Similarly, Cipolletta et al. (2019) demonstrated that psychoeducational support groups effectively improved anxiety, depression, and overall psychological well-being in breast cancer patients and their caregivers (Cipolletta et al., 2019). These interventions typically aim to prepare patients for symptom management, facilitate post-operative recovery, promote self-care practices, enhance perceived control over the illness experience, and support emotional regulation.

However, the evidence regarding psychoeducational interventions’ effectiveness remains mixed. While some studies have demonstrated significant benefits for anxiety and quality of life, their impact on depression and treatment adherence has been less consistent (Mustikaningsih et al., 2024). Also, research suggests that psychoeducational interventions may demonstrate limited effectiveness when implemented in isolation, particularly for patients with elevated baseline psychological symptoms. Guarino et al. (2020), in a systematic review and meta-analysis comparing cognitive-behavioral therapy (CBT), supportive-expressive therapy, and psychoeducational treatments, found that while psychoeducational interventions showed beneficial effects, they demonstrated smaller effect sizes compared to more structured therapeutic approaches, particularly in reducing anxiety and depression (Guarino et al., 2020). Furthermore, a recent randomized controlled trial comparing psychoeducational interventions and CBT in breast cancer patients found improvements in both intervention and control groups, but no statistically significant differences between groups (Ardizzone et al., 2022). In addition, passive educational interventions, such as providing written materials alone, have been shown to lack the beneficial effects of structured, interactive psychoeducational programs delivered within group or individualized formats.

Although psychoeducational programs may be strategically important to implement within Breast Units, their effectiveness as standalone interventions remains limited, suggesting the need for more comprehensive psychological approaches.

### 1.6 Eye movement desensitization and reprocessing

Eye movement desensitization and reprocessing (EMDR) therapy is grounded in the Adaptive Information Processing (AIP) model, which posits that psychological distress arises when traumatic experiences overwhelm the brain’s innate capacity to process information adaptively (Hase et al., 2017; de Jongh et al., 2019). According to the AIP model, when traumatic events occur—such as cancer diagnosis and subsequent surgical procedures—the distressing experience may be stored in state-specific form within isolated memory networks, along with associated distorted cognitions, intense emotions, and physiological sensations (Shapiro, 2017). These dysfunctionally stored memories remain unintegrated with adaptive memory networks containing more balanced perspectives and coping resources. When triggered by internal or external cues, these isolated memory networks can activate the original perceptions, emotions, and somatic experiences, manifesting as intrusive thoughts, avoidance behaviors, and hyperarousal symptoms characteristic of post-traumatic stress (Solomon and Shapiro, 2008).

The therapeutic mechanism of EMDR involves bilateral stimulation—typically eye movements, but also including auditory tones or tactile tapping—which is hypothesized to facilitate access to and reprocessing of these maladaptively stored memories (Stickgold, 2002; van den Hout and Engelhard, 2012; Landin-Romero et al., 2018). The working memory hypothesis suggests that performing bilateral stimulation while simultaneously attending to traumatic memories taxes working memory capacity, reducing the vividness and emotional intensity of the recalled experience (Solomon and Shapiro, 2008; Gunter and Bodner, 2009; Oren and Solomon, 2012; Shapiro, 2012; Wadji et al., 2022). This dual attention task enables the dysfunctionally stored memory to be accessed in a less overwhelming manner, facilitating its integration into adaptive memory networks and promoting symptom resolution (de Jongh et al., 2024).

To date, EMDR is a technique that has become increasingly established in the world of psychological and medical care, being progressively manualized, studied, and applied to countless psycho(patho-)logical contexts (de Jongh et al., 2019, 2024; Hase, 2021; Laliotis et al., 2023). Indeed, EMDR has proven to be a particularly effective technique (Davidson and Parker, 2001) not only for the treatment of trauma (Jeffries and Davis, 2013; de Jongh et al., 2019; Wright et al., 2024), but also for the treatment of depression (Sepehry et al., 2021), anxiety (Gauvreau and Bouchard, 2008; Faretta and Dal Farra, 2019), panic (Faretta, 2013; Faretta and Leeds, 2017), eating disorders (Hatoum and Burton, 2024; Rossi E. et al., 2024), some facets of personality disorders (Slotema et al., 2019).

Furthermore, EMDR has been increasingly applied in health psychology contexts, showing promising results for medically unexplained symptoms (Staton et al., 2023), chronic pain and fibromyalgia (Gerhardt et al., 2016; Tefft and Jordan, 2016; Vock et al., 2024), and psychological sequelae in patients with chronic medical conditions (Driessen et al., 2024) such as diabetes (Sheikhi et al., 2020), respiratory difficulties (Mooren et al., 2022), cardiac events (Arabia et al., 2011), and cardiovascular issues (Chen et al., 2025).

#### 1.6.1 EMDR in oncological settings

Concurrently, considering the traumatic impact of cancer, a growing body of scientific literature has demonstrated the efficacy of Eye Movement Desensitization and Reprocessing (EMDR) techniques in reducing symptoms of distress, anxiety, depression, and post-traumatic stress symptomatology in cancer patients (Carletto and Pagani, 2016; Faretta and Borsato, 2016; Carletto et al., 2019; Sepehry et al., 2021; Bates et al., 2023; Ciappina et al., 2024; Zucchetti et al., 2024).

A systematic review of EMDR therapy in cancer patients identified seven studies involving 140 cancer patients and concluded that, despite methodological limitations, available data suggest that EMDR could be a promising treatment for psychological distress in patients with cancer (Portigliatti Pomeri et al., 2021). The review found that across all analyzed studies, EMDR therapy was judged adequate in reducing symptoms of psychological distress, whether the diagnosis concerned PTSD or the broader anxious and depressive disorder spectrum (Portigliatti Pomeri et al., 2021). Comparative studies have demonstrated advantages of EMDR over other therapeutic approaches. A pilot randomized controlled trial comparing EMDR with CBT in oncology patients with PTSD found that for cancer patients in the follow-up stage, the absence of PTSD after treatment was associated with a significantly higher likelihood of receiving EMDR rather than CBT (Jarero et al., 2015).

Eye movement desensitization and reprocessing interventions have also been successfully adapted for group settings in oncological populations. Research on the EMDR Group Traumatic Episode Protocol (G-TEP) (Robinson and Kaptan, 2023) demonstrated statistically significant reductions in PTSD symptoms, with no serious adverse effects reported, suggesting that EMDR group protocols may be effective and safe in the psychological treatment of cancer patients (Jarero et al., 2015). Studies examining EMDR Integrative Group Treatment Protocol (IGTP) with female cancer patients have similarly shown significant reductions in PTSD symptoms related to cancer diagnosis and treatment (Jarero et al., 2015, 2016).

#### 1.6.2 The recent traumatic episode protocol (R-TEP)

Specifically, the EMDR R-TEP (Recent Traumatic Episode Protocol) (Shapiro and Laub, 2008; Shapiro, 2014) was designed for timely intervention in response to recent traumatic events – such as cancer diagnosis and/or subsequent surgical intervention (Clarke, 2023). This protocol has been shown to be effective in significantly reducing the psychological symptomatology previously described (Yurtsever et al., 2022; Torres-Giménez et al., 2024). Moreover, EMDR R-TEP was shown to be effective in containing the onset of post-traumatic symptomatology, especially following potentially life-threatening medical events (Shapiro, 2014; Bates et al., 2023).

The R-TEP protocol represents an early intervention approach within the EMDR framework, specifically developed for addressing recent traumatic experiences before they become consolidated into chronic post-traumatic symptomatology. Research has demonstrated its utility in critical illness survivors, including those recovering from intensive care, where it has been successfully implemented even via remote delivery (Bates et al., 2023). The protocol’s focus on early intervention is particularly relevant for breast cancer patients undergoing surgery, as it provides an opportunity to address traumatic responses during the immediate post-surgical period when psychological support may be most crucial for preventing long-term distress.

### 1.7 Research gaps and study rationale

Despite the growing evidence supporting EMDR interventions in oncological settings, research specifically examining the R-TEP protocol in breast cancer patients during the immediate post-surgical period remains limited. Furthermore, most existing studies have not employed rigorous randomized controlled trial designs comparing EMDR-based interventions with both active control conditions (such as psychoeducation) and treatment as usual approaches. This represents a significant gap in the literature, as understanding the relative efficacy of different psychological interventions is essential for optimizing clinical practice in oncological settings.

Additionally, while both psychoeducational interventions and EMDR have shown promise individually, there is limited research comparing their effectiveness within the same study population and examining their impact across a comprehensive range of psychological variables including anxiety, depression, distress, hopelessness, post-traumatic symptoms, and psychological well-being. Such comprehensive evaluation is necessary to identify which intervention modalities produce the greatest benefits for breast cancer patients and on which specific psychological dimensions.

### 1.8 Study aims and hypotheses

The present research project aims to implement, refine, and evaluate the efficacy of a psychological intervention supporting women affected by breast cancer who have undergone mastectomy or quadrantectomy. To achieve this objective, in addition to the standard psychological support intervention (treatment as usual; TAU), innovative integrated interventions will be applied, including psychoeducational interventions and interventions utilizing the potential of the EMDR R-TEP protocol.

Through a solid experimental methodology implemented as a randomized controlled trial (RCT), the project aims to identify the most effective intervention modalities for providing integrated psychological support to women with breast cancer. In a broader perspective, the study aims to provide a significant contribution not only to the scientific literature in the field but also to clinical practice, contributing to the optimization of clinical practices in oncology.

Specifically, the primary objective of the research is threefold:

1. To evaluate over time whether the EMDR R-TEP protocol produces clinically and statistically significant improvements in psychological variables of interest, including anxiety, depression, distress, hopelessness, traumatic impact of cancer diagnosis, psychological well-being, and global psychological symptomatology;
2. To assess the relative efficacy of the EMDR R-TEP protocol by analyzing differences in psychological variable improvements compared with the group receiving psychoeducational intervention and a control group receiving routine standard psychological support treatment (TAU);
3. To evaluate on which psychological variables the EMDR R-TEP protocol is capable of producing the greatest changes in terms of well-being, post-traumatic symptomatology, and stability over time.

It is hypothesized that the EMDR R-TEP protocol, combined with standard psychological support, will demonstrate superior efficacy in significantly reducing levels of distress, post-traumatic symptoms, and other psychological symptomatology (e.g., anxiety and depressive symptoms), compared to both TAU alone and TAU in addition to psychoeducation. The relative efficacy over time of the different treatments will be assessed through retest of the questionnaires identified in the assessment battery.

## 2 Methods and analysis

### 2.1 Study design

**Figure 1:**
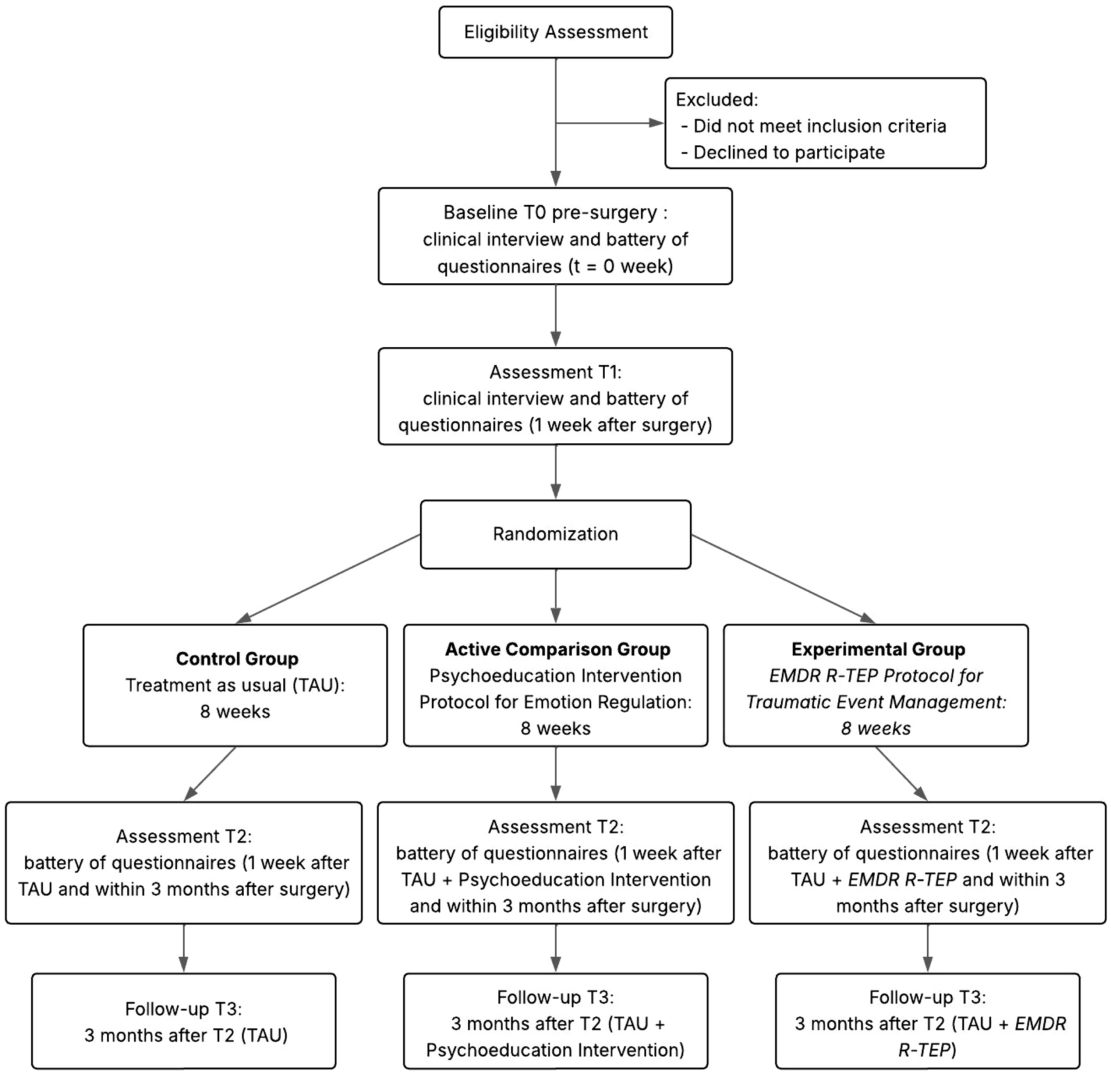
Flowchart.

This study employs a prospective, longitudinal, single-blind RCT design with three primary assessment timepoints (T0, T1, T2) and a long-term follow-up assessment (T3), and experimental psychological intervention with random allocation of participants across three groups (Figure 1): (A) Control group (G1): Treatment as usual (TAU) receiving standard psychological support; (B) Active control group (G2): TAU plus psychoeducational intervention; (C) Experimental group (G3): TAU plus EMDR R-TEP protocol.

The study follows a monocentric organizational structure, conducted within a single Breast Unit affiliated with ASST Rhodense. This investigation pursues non-commercial (non-profit) aims, solely for scientific and clinical purposes. The study protocol conforms to Consolidated Standards of Reporting Trials (CONSORT) guidelines (Schulz et al., 2010).

### 2.2 Participants

#### 2.2.1 Recruitment and setting

Breast cancer patients will be recruited continuously from the Breast Unit of the Oncology Department at ASST Rhodense (Milan, Italy). Recruitment is integrated within the standard clinical pathway of the Breast Unit. During routine outpatient consultations, medical staff will identify potentially eligible patients, conduct a preliminary screening of medical inclusion/exclusion criteria (e.g., absence of metastatic disease), and refer suitable candidates to the clinical psychology service. Clinical psychologists/psychotherapists will then conduct a psychological screening to verify remaining eligibility criteria (e.g., absence of severe psychiatric conditions, cognitive capacity to provide informed consent), provide information about study objectives and procedures, and obtain written informed consent. Patients will be informed of their right to withdraw at any time without consequences for their clinical care. The recruitment period is estimated at approximately 6 months.

#### 2.2.2 Inclusion criteria

Participants will be eligible if they: (1) are biologically female; (2) are aged between 18 and 80 years; (3) Have received first diagnosis of breast cancer; (4) Have scheduled surgical intervention (mastectomy or quadrantectomy); (5) Demonstrate excellent comprehension of Italian language; (6) Provide voluntary consent to participate in the study.

#### 2.2.3 Exclusion criteria

Participants will be excluded if they: (1) present with metastatic disease; (2) have cognitive impairment compromising understanding of informed consent and/or assessment instruments; (3) have history of severe psychiatric diagnosis with current psychotropic medication use; (4) presence of pre-existing PTSD unrelated to oncological illness; (5) clinically significant dissociative symptoms as indicated by (A) a clinical psychological examination and/or (B) a score higher than 30 on the Dissociative Experiences Scale-II (DES-II; Carlson and Putnam, 1993) – as severe dissociation may interfere with standard EMDR protocols and require adapted treatment approaches (van der Hart et al., 2006; ISSTD, 2011; Leeds et al., 2023).

#### 2.2.4 Sample size calculation

An *a priori* power analysis was conducted to calculate the minimum sample size required for this study, following established statistical procedures (Perugini et al., 2018; Taccini et al., 2022). The G*Power software (V.3.1.9.2) (Faul et al., 2007, 2009) was utilized to determine the required sample size for repeated measures analysis of variance (ANOVA) with within-between interaction. The research protocol involves four assessment timepoints, with treatment condition as between-group variable (TAU vs. TAU + psychoeducation vs. TAU+R-TEP) and time as within-subject variable (T0, T1, T2, and T3; see dedicated section).

Given the absence of prior studies implementing this specific intervention protocol, conservative a priori parameters were adopted. These were informed by examining previous studies utilizing similar interventions (though not identical), comparable target samples, and similar constructs. The effect size *f* was set to 0.15 (small-to-medium effect size) and the correlation among repeated measures was set to 0.35. Type I error rate probability (*α*) was set to 0.05 (two-sided), and power (1-*β*) was set at 0.80 (Cohen, 1988; Eid et al., 2017; Perugini et al., 2018).

G*Power analysis indicated 80% probability of correctly rejecting the null hypothesis of no significant group-by-time interaction effect with an overall sample of 102 participants (approximately 34 subjects per group). Based on average patient flow to the unit, to account for an anticipated attrition rate of approximately 20%, the study aims to recruit at least 122 patients, ensuring adequate statistical power for the planned analyses.

The three groups will each comprise a minimum of 34 patients: (A) Control group (G1): Patients receiving standard routine psychological support (TAU); (B) Active control group (G2): Patients receiving weekly psychoeducation session plus TAU; (C) Experimental group (G3): Patients receiving EMDR R-TEP intervention plus TAU.

This sample size is highly feasible considering service patient flow while simultaneously permitting statistical analyses generating reliable estimates.

#### 2.2.5 Randomization and blinding

The study employs a single-blind RCT design. Participant allocation to one of the three groups (TAU vs. TAU + psychoeducation vs. TAU+R-TEP) will follow rigorous procedures ensuring allocation concealment.

First, each participant will be assigned a unique identification code ensuring individual anonymity. Second, group allocation will be conducted through dedicated web-based software (Randomization.com) (Randomization, 2025) by two independent persons meeting the following requirements: (1) external to the project; (2) unaware of study objectives; (3) with no access to participant information; and (4) without mutual contact. If any condition is not met, these individuals will be replaced. This procedure ensures proper allocation concealment. Regarding blinding, participants are blinded to group assignment rationale, while treating clinicians are necessarily aware of intervention type given the nature of psychological treatments. Participants will be assigned to conditions within seven working days from baseline assessment.

### 2.3 Interventions

All three groups will receive psychological sessions twice weekly, conducted individually, in-person, with duration between 60–90 min.

#### 2.3.1 Control group (G1): treatment as usual (TAU)

Patients in the control group will receive unstructured psychological support sessions (without pre-established protocol) aimed at: reviewing individual history before and after disease discovery; exploring emotional experiences related to illness; identifying and strengthening individual resources; reducing psychological risk factors; promoting illness acceptance; and enhancing adherence to medical prescriptions and treatments. This approach reflects standard clinical practice in psycho-oncology settings and serves as the reference condition against which structured interventions are compared. TAU does not involve specific psychoeducation protocols or EMDR. However, if ongoing clinical monitoring during the therapeutic course reveals that a patient assigned to TAU would benefit more from a structured intervention (i.e., psychoeducation or EMDR R-TEP), clinicians will prioritize patient wellbeing and provide the most appropriate treatment. In such cases, the participant would be excluded from statistical analyses to maintain methodological rigor – as treatment crossover would violate the original randomized group assignment and compromise between-group comparability.

#### 2.3.2 Active control group (G2): TAU plus psychoeducational intervention

Patients in the active control group will participate in two individual sessions weekly combining psychological support with psychoeducational protocol. The primary objective of psychoeducation sessions is providing information regarding psychological aspects and experiences of oncological illness, with specific focus on post-traumatic aspects related to diagnosis, subsequent surgical intervention, and body image modification. The secondary objective involves helping patients recognize and structure functional emotion regulation strategies, including tools for improving observation and description abilities regarding their emotions in the present moment.

The psychoeducational intervention was developed in accordance with core components identified in the psycho-oncology literature (Al-Alawi et al., 2022; Setyowibowo et al., 2022), including information provision, emotional expression, coping skills training, and social support (Ratti et al., 2017; Panzeri et al., 2022; Taccini et al., 2022, 2025).

The 8-week individual intervention is structured as follows: (1) psychoeducation on cancer diagnosis and psychological reactions; (2) psychoeducation on trauma and post-traumatic responses; (3) emotional recognition and validation; (4) emotion regulation strategies I (grounding, breathing, relaxation techniques); (5) emotion regulation strategies II (cognitive reframing of illness-related thoughts); (6) body image and femininity after surgery; (7) personal resources and social support; (8) integration and future planning. Each session lasts 60–90 min and follows a semi-structured format allowing clinical flexibility while maintaining content consistency across participants. A description of the intervention structure is provided in Supplementary Table S1.

Regarding potential adverse events, psychoeducational interventions may occasionally elicit emotional activation or transient distress when addressing trauma-related content or body image issues. Clinicians will monitor for such reactions throughout the therapeutic course. Management of adverse events will be handled at clinical discretion – given that the heterogeneity of possible reactions precludes a standardized intervention protocol. All adverse events will be documented in the study database. Should a participant experience recurrent or severe adverse reactions compromising their clinical stability, or when clinically indicated, they will be reassigned to TAU and excluded from statistical analyses – as treatment crossover would violate the original randomized group assignment and compromise between-group comparability.

#### 2.3.3 Experimental group (G3): TAU plus EMDR R-TEP

Patients in the experimental group will participate in two individual weekly sessions receiving psychological support plus EMDR R-TEP protocol administration. While sharing the supportive elements of TAU, this condition adds the specific trauma-focused R-TEP protocol, allowing evaluation of the incremental benefits of EMDR-based intervention compared to both unstructured support (G1) and structured psychoeducation (G2). According to guidelines, the EMDR R-TEP intervention is structured in eight phases (Shapiro and Laub, 2008):

1. *History taking and case conceptualization*: Special attention to recent cancer diagnosis, traumatic experiences during medical investigations and diagnostic procedures, emotion-focused coping modalities and self-regulation capacity, vulnerability to traumatic experiences, triggers eliciting disease-related episodes, significant relational factors, and internal/external resource analysis.
2. *Patient preparation* for subsequent protocol phases, with specific attention to patient safety and containment.
3. *Assessment and target selection*: Targets relate to present dimension or recent past (i.e., cancer diagnosis). This phase utilizes subjective distress evaluation measured with Subjective Units of Distress (SUD) scale, ranging 0–10, where 0 represents distress absence and 10 represents highest distress level experienced.
4. *Desensitization and reprocessing* through bilateral stimulation.
5. *Installation* of positive cognition using bilateral stimulation sets.
6. *Body scan*.
7. *Closure*: If SUD remains > 2, an additional session will be considered.
8. *Reevaluation*: Each session begins with patient distress reevaluation following prior EMDR session.

Treatment fidelity for the EMDR R-TEP intervention will be ensured through the following procedures: (a) all therapists delivering the R-TEP protocol are trained and certified EMDR practitioners; (b) therapists will follow the manualized eight-phase R-TEP protocol as described by Shapiro and Laub (2008); (c) regular clinical supervision will be provided throughout the study duration to monitor protocol adherence and address implementation challenges. Formal independent fidelity assessment (e.g., session recording and external rating) was not included due to resource constraints, representing a limitation that should be addressed in future replication studies.

Regarding potential adverse events, EMDR-based interventions may occasionally elicit transient distress, emotional activation, or temporary symptom exacerbation during trauma reprocessing (van der Hart et al., 2006). Trained clinicians will monitor for such reactions throughout the therapeutic course, including transient dissociative responses, heightened anxiety, or emotional dysregulation. Management of adverse events will be handled at clinical discretion, or – when available – in line with EMDR guidelines. All adverse events will be documented in the study database. Should a participant experience recurrent or severe adverse reactions compromising their clinical stability, or when clinically indicated, they will be reassigned to TAU and excluded from statistical analyses – as treatment crossover would violate the original randomized group assignment and compromise between-group comparability.

### 2.4 Procedure

#### 2.4.1 Enrollment and informed consent

Following Breast Unit referral, patients will undergo individual interviews with healthcare professionals (clinical psychologists/psychotherapists) who assess study eligibility based on inclusion/exclusion criteria. Eligible individuals will receive detailed study information to obtain informed consent. Participants will be informed that: (i) participation is voluntary; (ii) refusal or withdrawal has no repercussions on care access or quality; (iii) withdrawal is permitted anytime with data deletion option; (iv) no economic compensation is provided; (v) group assignment does not influence psychological support quality.

Consenting participants will proceed to subsequent research phases (assessment). All procedures conform to the Diagnostic-Therapeutic Care Pathway (PDTA) in effect within the Service (PDTA ASST-Rhodense n.6 Rev. 04 approved 27.01.2025).

#### 2.4.2 Timeline and assessments

According to the flowchart of the study (Figure 1), participants will undergo six different steps.

1. *Baseline T0* (post-diagnosis, pre-hospitalization, pre-surgery): Participants will complete clinical interview and self-report psychological assessment battery.
2. *Surgery:* participants will undergo breast surgery.
3. *Follow-up T1* (within 1 week post-surgery): Participants will complete clinical interview and repeat self-report instruments to evaluate temporal changes.
4. *Group assignment:* Following T1 assessment, participants will be randomly assigned to one of three groups, then follow their respective 8-week psychological intervention.
5. *Follow-up T2* (psychological intervention completion, within 3 months post-surgery): All participants will complete the psychological assessment battery again to evaluate temporal changes.
6. *Follow-up T3* (within 3 months post-psychological intervention): All participants will complete the psychological assessment battery again.

Participants who withdraw (dropout) from the study will not have their data considered in analyses. However, these individuals will continue receiving treatment benefits from their assigned condition. To ensure complete and optimal data analysis, questionnaires presenting missing data will be excluded from the study (Pozzar et al., 2020) – though participants will continue benefiting from assigned treatment.

### 2.5 Outcome measures

In accordance with study objectives, this research protocol assesses a comprehensive range of psychological outcomes relevant to breast cancer patients, including affective symptoms, trauma-related distress, body image, self-esteem, emotion regulation difficulties, perceived social support, and global psychological functioning.

An anamnestic form will collect sociodemographic variables (age, marital status, education, family composition, employment status, current intimate relationship information), immigration status, alcohol consumption, substance abuse, and notable life events (separation, bereavement, etc.). Additionally, beyond standard clinical interview, temporal changes will be monitored using validated Italian-language self-report instruments.

All assessments will be administered at baseline (T0), after surgical intervention (T1), after completion of the psychological intervention (T2), and at 3-month follow-up from the complention of the psychological intervention (T3) - to evaluate long-term changes.

#### 2.5.1 Primary outcomes

Based on the theoretical framework previously outlined, primary outcomes were selected to assess the core psychological constructs targeted by the intervention: psychological distress and post-traumatic symptomatology following breast cancer diagnosis and surgery.

##### 2.5.1.1 Psychological distress inventory – revised (PDI-R)

The PDI-R (Rossi et al., 2022) is a self-report questionnaire specifically created for distress measurement in oncological settings. Comprising 8 items, it is suitable for medical setting test batteries. Beyond providing total distress score, clinical cut-offs and normative scores divided by sex and age have been developed. The instrument’s psychometric properties proved excellent.

##### 2.5.1.2 Post-traumatic symptom questionnaire (PTSQ)

The PTSQ (Rossi et al., 2024b) is a 12-item questionnaire developed to evaluate post-traumatic stress symptoms (PTSS) and traumatic event impact across three components: (i) intrusion, (ii) avoidance, and (iii) hyperarousal. Given its administration agility, the PTSQ particularly suits PTSS measurement across diverse contexts (Rossi et al., 2024c) compared with lengthier instruments (Rossi et al., 2024a). Beyond providing scores for the three mentioned components, the PTSQ yields total score with developed clinical cut-offs (Rossi et al., 2024a). Psychometric properties proved excellent.

#### 2.5.2 Secondary outcomes

Secondary outcomes were selected to capture additional dimensions of psychological functioning relevant to breast cancer patients, including anxiety and depressive symptomatology, hopelessness, body image, and global psychological wellbeing.

##### 2.5.2.1 Hospital anxiety and depression scale (HADS)

The HADS (Zigmond and Snaith, 1983; Norton et al., 2013) is among the world’s most utilized self-administered tests for evaluating anxious and depressive symptomatology in medical/organic settings. The HADS comprises 14 items, yielding two scores: anxiety scale and depression scale. In the present study, the two subscales will be used separately to assess anxiety and depressive symptomatology as distinct outcomes. The Italian version (Annunziata et al., 2011) demonstrated excellent psychometric properties.

##### 2.5.2.2 Beck hopelessness scale (BHS)

The BHS (Beck et al., 1985) is a self-report test specifically created for evaluating hopelessness feelings – a key psychological element in treating complex medical conditions like depression. It comprises 20 dichotomous items (true/false). The Italian version (Iliceto and Fino, 2015) showed excellent psychometric properties.

##### 2.5.2.3 Body image scale (BIS)

The BIS (Hopwood et al., 2001; Melissant et al., 2018) is a self-administered questionnaire specifically developed for body image evaluation in patients with medical/organic and oncological pathologies. Comprising 10 items, it yields a single total score. The Italian version (Cheli et al., 2016) demonstrated good psychometric properties.

##### 2.5.2.4 Difficulties in emotion regulation scale – 8 (DERS-8)

The DERS-8 (Penner et al., 2023) is an 8-item measure assessing emotion regulation difficulties (Gratz and Roemer, 2004) as a single dimension. Items cover difficulties in acceptance, goal-directed behavior under distress, impulse control, and access to effective regulation strategies. The Italian version (Rossi et al., 2025) demonstrated excellent psychometric properties.

##### 2.5.2.5 Rosenberg self-esteem scale (RSES)

The RSES (Rosenberg, 1965) is a widely used 10-item measure assessing global self-esteem in general and clinical populations, defined as an individual’s overall evaluation of their own worth. The Italian version (Prezza et al., 1997) demonstrated good psychometric properties.

##### 2.5.2.6 Psycho-social support scale (PSSS)

The PSSS (Panzeri et al., 2022) is a brief 4-item self-report measure assessing perceived psycho-social support, capturing the extent to which individuals feel supported by their social environment. Higher scores indicate greater perceived support. The scale demonstrated good psychometric properties.

##### 2.5.2.7 Clinical outcomes in routine evaluation – outcome measure (CORE-OM)

The CORE-OM (Evans et al., 2000) is among the world’s most utilized instruments for evaluating general symptomatology and suicidal risk. It examines four distinct psychological functioning domains: (1) subjective wellbeing; (2) problems and symptoms; (3) social and psychological functioning; and (4) self/other-harm risk. The Italian version (Palmieri et al., 2009) demonstrated good psychometric properties.

### 2.6 Statistical analysis

Statistical data analyses will be performed using R software (R Core Team, 2017). Throughout data collection duration, *in itinere* statistical analyses will verify data quality and potential bias absence. Regarding missing data management, participants who discontinue the study (dropouts) will be excluded from analyses due to concerns about response validity. Similarly, assessment batteries with more than 10% missing items will be considered invalid and excluded. For remaining missing data within valid batteries, appropriate handling methods (e.g., multiple imputation, full information maximum likelihood) could be employed depending on missing data patterns and mechanisms.

Preliminary analyses will verify group homogeneity regarding relevant variables (age, comorbidity, social support, etc.) and possible intervening variable influence (covariates). If deemed appropriate and/or if significant heterogeneity is detected between groups, such variables will be included as covariates in the analysis models. Preliminary analyses will also verify psychometric instruments’ internal consistency (McDonald’s omega) (McDonald, 1999).

Both group-level aggregate analyses and individual change-focused evaluations will be employed. At group level, longitudinal changes will be examined using appropriate linear models for repeated measures data (e.g., linear mixed models or repeated measures ANOVA, or Bayesian approaches), depending on data characteristics such as distribution, missing data patterns, and sphericity assumptions (Howell, 2013; Darlington and Hayes, 2017; van den Bergh et al., 2023). Also, if appropriate, multilevel analysis – random slopes and/or random intercepts – may be implemented in the models (Hox et al., 2017; Heck and Thomas, 2020; Finch and Bolin, 2024). Model selection will be guided by standard diagnostic procedures. All models will test the interaction between time and intervention group – and where appropriate – controlling for individual variability and potential covariates identified in preliminary analyses.

Additionally, between-group comparisons at each timepoint may be performed using appropriate statistical tests depending on data distribution (e.g., one-way ANOVA or Kruskal-Wallis). If appropriate, post-hoc pairwise comparisons (R-TEP vs. TAU, R-TEP vs. psychoeducation, psychoeducation vs. TAU) will be conducted to identify specific group differences. Within-group comparisons across timepoints (e.g., T0 vs. T1, T1 vs. T2) may also be examined to evaluate change trajectories within each condition. Effect sizes (e.g., Cohen’s d for pairwise comparisons, partial η^2^ for omnibus tests) will be reported to facilitate clinical interpretation (Cohen, 1988). If necessary, to control for Type I error inflation due to multiple testing, the False Discovery Rate (FDR) correction procedure will be applied to the primary outcomes (Benjamini and Hochberg, 1995; Storey, 2002). Secondary outcomes will be analyzed as exploratory. Moreover, if Bayesian approaches are employed, Bayes Factors will be reported to quantify evidence strength for both alternative and null hypotheses (Greenland and Poole, 1994; Kass and Raftery, 1995; Lavine and Schervish, 1999; Held and Ott, 2018).

At individual level, parameters identifying significant changes both statistically (e.g., Reliable Change Index) and/or clinically (e.g., Minimal Clinically Important Difference) could be calculated (Maassen, 2000, 2001; Turner et al., 2010). Additionally, when available, individual scores will be compared with normative scores (expressed in Z or T points) to evaluate symptom severity relative to reference population (Howell, 2013; Tabachnick and Fidell, 2019).

### 2.7 Ethics approval and trial registration

The study protocol has been approved by the Ethics Committee Milano Area 3 of ASST Grande Ospedale Metropolitano Niguarda (protocol n° 5980), which is responsible for ethical review of clinical trials and observational studies conducted across all hospital facilities of ASST Rhodense. The study will be conducted in accordance with the principles outlined in the Declaration of Helsinki and follows Good Clinical Practice (GCP) guidelines.

The protocol will be pre-registered with the BMC ISRCTN registry (ISRCTN Registry; ID: ISRCTN17036586) in conformity with best practices in clinical psychology research. Any relevant protocol modifications will be reported to the ethics committee and updated in the trial registry, with changes documented in subsequent publications.

## 3 Discussion

Despite widespread recognition of the importance of psychological interventions in oncology, substantial barriers prevent many patients from receiving adequate psychological support to manage and address the traumatic impact of diagnosis and illness (Green et al., 1997; Rossi et al., 2024a) – further improving psychological well-being. The resulting gap between need and service provision represents a critical healthcare challenge requiring innovative solutions.

This research addresses a significant gap in evidence-based psychological care for breast cancer patients. If effective, the EMDR R-TEP protocol could be systematically implemented in Breast Units as standard practice, providing timely psychological intervention to reduce trauma-related psychological morbidity following cancer diagnosis and surgery. This would represent meaningful advancement in comprehensive breast cancer care, potentially improving both psychological outcomes and medical treatment adherence.

Beyond individual patient benefit, demonstrating efficacy of a brief, structured psychological intervention may influence healthcare resource allocation, supporting expansion of psychological services within oncological settings. The non-pharmacological nature of the intervention also addresses the ethical imperative to provide psychological relief without adding medication burden to patients already facing complex medical regimens.

### 3.1 Expected psychological outcomes

Based on the theoretical framework outlined in the Introduction, both the psychoeducational intervention (G2) and the EMDR R-TEP protocol (G3) are expected to demonstrate superior efficacy compared to TAU (G1), as both structured interventions specifically target core constructs relevant to this population. However, greater improvement is expected for individuals assigned to the EMDR R-TEP protocol at all timepoints, given its specific focus on trauma reprocessing through bilateral stimulation – directly addressing the traumatic underpinnings of psychological symptomatology in breast cancer patients following diagnosis and surgery. While the difference between the two structured interventions may be less pronounced at the earlier timepoint (T2), the superiority of EMDR R-TEP is hypothesized to be most evident at long-term follow-up (T3), in line with the trauma-focused literature and the mixed efficacy results for psychoeducational interventions.

### 3.2 Innovation and expected impact

This study protocol represents a significant advancement in the intersection of psycho-oncology and trauma-focused psychological interventions. The B.R.E.A.S.T. study addresses multiple critical gaps in current research and clinical practice, with potential to transform psychological care delivery within Breast Units.

#### 3.2.1 Scientific innovation

The primary scientific innovation lies in this being the first randomized controlled trial specifically evaluating EMDR R-TEP protocol for breast cancer patients following surgical intervention. While previous research has examined standard EMDR protocols in oncological populations (Carletto and Pagani, 2016; Torres-Giménez et al., 2024) no studies have systematically investigated the R-TEP protocol’s unique utility for addressing acute traumatic impact of recent cancer diagnosis and surgery. This represents a fundamental contribution to the trauma treatment and psycho-oncology literature.

The three-arm comparative design enables rigorous evaluation not merely of whether R-TEP is effective, but whether it offers meaningful advantages beyond current standard care (TAU) and established psychoeducational approaches. This comparative framework provides clinically actionable evidence regarding relative intervention efficacy—information essential for evidence-based practice guidelines and healthcare resource allocation decisions.

The comprehensive, multi-dimensional assessment battery employing validated, oncology-specific instruments permits nuanced understanding of intervention effects across psychological domains including distress, anxiety, depression, post-traumatic symptomatology, hopelessness, body image, and global psychological functioning. This approach will illuminate not only whether R-TEP works, but specifically how it works and which psychological targets demonstrate greatest treatment responsiveness.

#### 3.2.2 Clinical innovation and feasibility

The protocol’s brevity and integration potential represents crucial innovation for clinical implementation. Unlike extended psychological interventions facing practical barriers in busy oncological settings, this time-limited protocol offers realistic integration within existing Breast Unit care pathways. The intervention timing—early post-surgical implementation—capitalizes on the critical window for preventing traumatic memory consolidation and chronic post-traumatic symptom development, embodying a preventive rather than merely reactive approach.

The standardized, manualized protocol enhances both research rigor and clinical disseminability. Clearly defined intervention components facilitate training, quality assurance, and replication across different Breast Unit settings. This contrasts with less structured psychological support approaches whose variable content may produce inconsistent outcomes.

#### 3.2.3 Healthcare system implications

Demonstrating that a brief, non-pharmacological intervention can meaningfully alleviate psychological burden carries significant healthcare system implications. Among the most relevant potential benefits is a reduced reliance on psychotropic medications, thereby avoiding polypharmacy in patients already managing complex medical regimens and the associated side effect burdens. Furthermore, effective early psychological intervention may decrease emergency psychiatric consultations and crisis interventions by preventing psychological deterioration before it reaches critical thresholds. Importantly, improved psychological functioning has been consistently linked to enhanced treatment adherence and better medical outcomes, given that psychological distress negatively impacts compliance and may adversely affect medical prognosis. From an economic perspective, brief and focused interventions offer considerable cost-effectiveness by preventing the need for more intensive and prolonged psychological treatments, while also reducing overall healthcare utilization typically associated with poorly managed psychological distress. Taken together, these potential systemic benefits provide compelling rationale for policy-level decisions regarding the systematic integration of psychological services within comprehensive cancer care.

#### 3.2.4 Broader scientific contributions

Beyond immediate clinical applications, this research contributes to theoretical understanding of trauma processing in medical populations. Cancer diagnosis and treatment represent unique traumatic experiences—anticipated, medicalized, often prolonged—differing from many trauma types (e.g., assault, accidents) for which EMDR was originally developed. Examining R-TEP efficacy in this context advances understanding of trauma treatment mechanisms and applicability across diverse traumatic experiences (Panzeri et al., 2023; Rossi et al., 2024a).

The study also contributes to biopsychosocial oncology models, providing evidence regarding psychological intervention impact on the integrated biological-psychological-social experience of cancer. Future analyses potentially incorporating physiological markers (if available) could shed light on the mind–body connections in cancer care.

### 3.3 Strengths and limitations

#### 3.3.1 Limitations

Several limitations of the present study should be acknowledged. First, the single-center design, with the study being conducted within a single Breast Unit, may limit generalizability to other settings with different organizational structures, patient populations, or resource availability; future multi-center trials would enhance external validity. Additionally, TAU is by nature an unstructured and non-manualized intervention; given the inherent variability of such approaches, this represents a potential source of heterogeneity that should be acknowledged. Second, as noted in the protocol, patient recruitment will require considerable time and coordination, potentially extending study duration beyond initial projections. Third, while patients are blinded to group assignment rationale, treating clinicians necessarily know intervention type, potentially introducing expectancy effects; however, standardized assessment instruments and statistical analysis by independent researchers mitigate this limitation. Fourth, post-intervention follow-up, while sufficient for detecting immediate treatment effects, does not capture long-term outcome stability; extended follow-up at 12 month would strengthen conclusions regarding intervention durability. Fifth, inclusion criteria (e.g., excluding metastatic disease, severe psychiatric conditions) ensure sample homogeneity for internal validity but may limit applicability to more complex or severely ill populations. Sixth, the exclusion of patients with current psychotropic medication use, while limiting generalizability, was implemented to establish internal validity in this initial efficacy trial. Testing the intervention in a homogeneous sample without pharmacological confounds allows for clearer attribution of observed effects to the psychological intervention itself. Once efficacy is established, future effectiveness studies should examine R-TEP in broader clinical populations, including patients with stable psychotropic medication regimens. Similarly, the exclusion of patients with pre-existing PTSD unrelated to oncological diagnosis and those with clinically significant dissociative symptoms was implemented to ensure sample homogeneity and treatment safety, as severe dissociation may interfere with standard EMDR protocols (van der Hart et al., 2006; ISSTD, 2011; Shapiro, 2017; Poli et al., 2023). Future studies should explore adapted R-TEP approaches for patients with complex trauma histories or dissociative features. Finally, while the protocol measures various outcome domains, it does not assess specific process or mechanism measures (e.g., trauma memory processing, emotion regulation mechanisms). This gap limits the ability to identify the active components of the intervention and understand how EMDR R-TEP produces its effects. Future studies incorporating process measures would enhance theoretical understanding.

#### 3.3.2 Strengths

The present study presents several methodological strengths. First, the randomized controlled trial design with active comparison groups represents gold-standard evidence generation, minimizing bias and enhancing confidence in findings. Second, multi-dimensional evaluation across validated instruments provides comprehensive assessment and detailed understanding of intervention effects and mechanisms. Third, sample size calculations ensure adequate statistical power to detect clinically meaningful effects, reducing false negative risk. Fourth, the manualized R-TEP protocol enhances internal validity, treatment fidelity, and future replicability through standardized intervention delivery. Fifth, the intervention addresses a genuine unmet clinical need with feasible implementation potential in real-world oncological settings, ensuring clinical relevance. Finally, early post-surgical implementation capitalizes on prevention opportunities rather than waiting for chronic symptom development, allowing for timely intervention.

### 3.4 Future research directions

This study establishes foundation for multiple future research avenues:

Extended follow-up studies could examine long-term outcome stability and delayed intervention effects, determining whether early R-TEP prevents later psychological complications.

Mechanism-focused research could investigate specific processes through which R-TEP produces effects, potentially incorporating neuroimaging, psychophysiological measures, or detailed process assessments during treatment sessions.

Comparative effectiveness research could examine R-TEP against other evidence-based trauma treatments (e.g., trauma-focused CBT, prolonged exposure) to determine optimal intervention selection.

Implementation science studies could evaluate real-world R-TEP integration in diverse Breast Unit contexts, examining barriers, facilitators, training requirements, and cost-effectiveness in routine practice.

Expanded populations could test R-TEP efficacy in other cancer types, patients with metastatic disease, or those with pre-existing psychological vulnerabilities, extending evidence base across cancer populations.

Moderator and predictor analyses could identify which patients benefit most from R-TEP versus alternative approaches, enabling personalized treatment matching.

Combined intervention studies could examine whether integrating R-TEP with other supportive interventions (e.g., physical rehabilitation, peer support, mindfulness programs) produces synergistic effects.

## 4 Conclusion

The B.R.E.A.S.T. study addresses critical gaps in psychological oncology care through rigorous evaluation of an innovative, theoretically-grounded, practically feasible intervention for breast cancer patients. By examining EMDR R-TEP protocol efficacy in this population, the research has potential to transform clinical practice, inform healthcare policy, advance scientific understanding of trauma treatment in medical contexts, and ultimately improve outcomes for women facing breast cancer diagnosis and treatment.

If hypotheses are supported, findings will provide compelling evidence for systematic R-TEP implementation as standard practice within Breast Units, contributing to comprehensive biopsychosocial cancer care. Regardless of specific outcomes, the study will generate valuable evidence informing the evolving field of psycho-oncology and establishing foundation for continued investigation of optimal psychological care for cancer patients.
