# Supplementary material for: B.R.E.A.S.T. Study protocol: Benefits of R-TEP EMDR protocol in Addressing diStress and Trauma in breast cancer patients
Source: Front Psychol. 2026 Jan 20;16:1759849. doi: 10.3389/fpsyg.2025.1759849 (PMC12866898; doi:10.3389/fpsyg.2025.1759849)
Supplement: Supplementary file 1 [file Data_Sheet_1.PDF]

Supplementary Table S1. Structure of the psychoeducational intervention for breast cancer patients.

Session	Title	Objectives	Key Contents	Techniques
1	Cancer diagnosis and psychological reactions	Normalize emotional responses to diagnosis; provide information on the psychological impact of oncological illness	Common psychological reactions to cancer diagnosis; relationship between physical illness and emotional distress; individual illness representations and beliefs	Psychoeducation; guided discussion; emotional validation
2	Trauma and post-traumatic responses	Provide information on trauma and post-traumatic reactions; normalize post-traumatic symptoms in the oncological context	Definition of psychological trauma; post-traumatic symptomatology (intrusion, avoidance, hyperarousal); distinction between normal reactions and pathological responses	Psychoeducation; normalization; guided discussion
3	Emotional recognition and validation	Facilitate identification and labeling of emotions; promote emotional acceptance and validation	Identification of prevalent emotions (fear, anger, sadness, guilt, shame); emotional vocabulary development; self-validation of emotional experiences	Emotional awareness exercises; reflective discussion; validation techniques
4	Emotion regulation strategies I	Introduce and practice physiological regulation techniques; provide tools for managing acute emotional activation	Grounding techniques; diaphragmatic breathing; progressive muscle relaxation; mindfulness-based present-moment awareness	Experiential practice; guided exercises; at-home practice planning
5	Emotion regulation strategies II	Introduce cognitive strategies for emotion regulation; identify and modify illness-related dysfunctional thoughts	Relationship between thoughts and emotions; common cognitive distortions in oncological patients; cognitive reframing of illness-related beliefs	Psychoeducation; cognitive restructuring exercises; guided discovery
6	Body image and femininity after surgery	Explore the impact of surgery on body image and sense of femininity; facilitate adjustment to bodily changes	Body image disturbances following mastectomy/quadrantectomy; mourning the pre-illness body; femininity and self-concept; pathway toward bodily re-appropriation	Reflective discussion; emotional processing; self-compassion exercises
7	Personal resources and social support	Identify and strengthen internal and external resources; address communication with family and social network	Personal coping resources; social support networks; communication strategies with family members and caregivers; managing relationships during illness	Resource mapping; communication skills; problem-solving
8	Integration and future planning	Consolidate learning; facilitate future planning and relapse prevention	Review of acquired skills and strategies; personal action plan development; identification of early warning signs; future perspectives and goal setting	Summary and integration; action planning; closure

Note. *Each session lasts 60-90 minutes and is delivered individually. The intervention follows a semi-structured format: session objectives and key contents are standardized across participants, while specific examples, exercises, and depth of exploration are adapted to individual patient needs and clinical presentation. Sessions are delivered by clinical psychologists/psychotherapists with training in psycho-oncology.*
